# Autism in Adults in Romania: Challenges in Diagnosis and Screening

**DOI:** 10.31083/AP39058

**Published:** 2025-10-28

**Authors:** Alexandra Dolfi, Darian Faur, Mihai-Rareș Scălcău, Andrei Chișcu, Cătălina Tudose

**Affiliations:** ^1^“Prof. Dr. Alexandru Obregia” Psychiatry Hospital, 041914 Bucharest, Romania; ^2^Doctoral School, “Carol Davila” University of Medicine and Pharmacy, 050474 Bucharest, Romania; ^3^Department of Psychology, West University of Timișoara, 300223 Timisoara, Romania; ^4^Romanian Association of Behavioral and Cognitive Therapy, 011277 Bucharest, Romania; ^5^“Carol Davila” University of Medicine and Pharmacy, 050474 Bucharest, Romania

**Keywords:** autism spectrum disorder (ASD), autism quotient (AQ), empathy quotient (EQ), psychometric properties, screening, Romanian

## Abstract

**Background:**

Due to the absence of validated screening tools for Autism Spectrum Disorder (ASD) in adults without intellectual or language deficits in Romania, clinicians often overlook ASD during evaluations, leading to frequent misdiagnoses. To screen for symptoms of comorbid pathologies in an ASD sample compared with a non-ASD sample using the Psychiatric Diagnostic Screening Questionnaire (PDSQ) and to establish cut-off scores for the Romanian-translated versions of the Autism Quotient (AQ) and Empathy Quotient (EQ).

**Methods:**

The study included 143 participants, 31 diagnosed with ASD and 112 from the general population. Both groups completed the PDSQ, AQ, and EQ. Analyses focused on the factorial structure, reliability, criterion validity, sensitivity, and specificity of the AQ and EQ, as well as correlations between AQ/EQ scores and PDSQ scores.

**Results:**

Higher AQ scores were associated with anxiety, trauma, and obsessive-compulsive disorder (OCD) symptoms. A cut-off score of 21 on the AQ accurately classified 100% of clinically diagnosed ASD participants and correctly identified 80% of non-ASD participants, yielding an overall accuracy of 84%. For the EQ, a cut-off score of 26 achieved the highest specificity while maintaining optimal sensitivity, with an overall accuracy of 88%. Both AQ and EQ demonstrated good internal consistency and reliability.

**Conclusion:**

The Romanian versions of the AQ and EQ are highly reliable screening tools for clinical use. Correlations between AQ scores and elevated anxiety, OCD, and trauma symptoms on the PDSQ highlight the importance of assessing ASD comorbidities during clinical evaluations.

## Main Points

1. Despite neurodiversity’s popularity over the last decade, many patients seek diagnosis confirmation abroad because Romania lacks tailored screening tools and diagnostic protocols for Autism Spectrum Disorder (ASD). The absence of ASD screening tools often causes specialists to overlook it during initial evaluations, leading to misdiagnosis.

2. This is the first study conducted on the Romanian population, which establishes a cut-off score for the Autism Quotient (AQ) and for the Empathy Quotient (EQ) on a sample of Romanian speakers (21 for AQ, 26 for EQ) by analyzing the statistical measures of AQ and EQ on a clinical (N = 31) and non-clinical sample (N = 112).

3. The AQ and EQ were correlated with the psychopathology measures of the Psychiatric Diagnostic and Screening Questionnaire (PDSQ) to establish the associated symptoms, without correlating symptom severity.

4. Our study concluded that the Romanian versions of the AQ and EQ are highly reliable, even though the cut-off scores obtained were smaller than those cited in the literature. This shows that further studies are necessary to revise the AQ and EQ screening tools, which have been used since 2001.

5. Higher scores for anxiety, obsessive-compulsive symptoms, and trauma were obtained on PDSQ in correlation with AQ, emphasizing that the comorbidities of ASD should receive greater attention during clinical evaluations.

## 1. Introduction

Autism spectrum disorder (ASD) is a neurodevelopmental disorder, which can be difficult to diagnose, especially in adults without cognitive and language deficits. In Romania, these adults are frequently misdiagnosed or go under the radar being undiagnosed for decades, until functionality problems appear due to stress factors or the appearance of other psychiatric or physical comorbidities which accentuate the symptomatology of ASD. Due to the lack of validated screening tools and diagnosis protocols, most clinicians in Romania do not consider ASD when they evaluate adults, leading to them receiving another diagnosis (e.g., anxiety, depression, obsessive-compulsive disorder (OCD)), which is, most of the time, a comorbidity of ASD, not the main diagnosis [1]. Another difficulty is the fact that parents or caregivers are unavailable most of the time at the time the adult requests an assessment, which is most of the time delayed, especially in adults with a higher intelligence quotient and good language abilities who develop effective coping strategies and present symptoms later in life [2, 3]. ASD is even harder to diagnose in women, who are later diagnosed than males [4], not only due to the fact that autism is higher prevalent in males [5] but also because women develop different coping strategies and the expression of their symptoms is more subtle, resembling more with anxiety and depression than with ASD [6, 7]. The comorbidities of ASD not only coexist with the primary condition but can also arise from ASD. Autistic adults are more vulnerable due to their difficulties in communication and adapting to society, making them more likely to experience isolation and bullying from their peers, which can lead to anxiety, depression, and trauma-related disorders [8, 9]*.* OCD and attention deficit and hyperactivity disorders are frequent comorbidities of ASD [10, 11], along with mood-related disorders, psychosis, and personality disorders [12].

Many adults seeking an ASD diagnosis are misdiagnosed with the conditions mentioned above, due to overlapping symptoms [13], leading to comorbidity being recognized as the primary diagnosis while ASD remains undiagnosed.

There are no studies in Romania up to date which show the comorbidities of ASD without intellectual and language deficits in adults. The aim of our study was to screen the associated symptoms of ASD in a clinical sample compared to the non-clinical sample using the Psychiatric Diagnosis and Screening Questionnaire (PDSQ), and to establish a cut-off score for the Autism Quotient (AQ) and Empathy Quotient (EQ) on the Romanian population. AQ and EQ are not validated on the Romanian population at the moment of data collection for the current study, but the psychometric properties of PDSQ have been assessed in both a clinical and non-clinical sample of Romanian speakers [14].

## 2. Materials and Methods

### 2.1 Participants and Procedure

Our initial sample was comprised of N = 145 participants. After the initial data inspection, two participants, one from each group, were removed because they were outliers. The final sample had N = 143 participants. Of these, 31 (21.7%) were participants diagnosed with ASD, while 112 (78.3%) were participants from the general population. We estimated our sample size using the pROC package in R [15], which allows power testing for receiver operating characteristic (ROC) curves. For a minimal expected area under the curve (AUC) = 0.7, at a standard α = 0.05 with 80%, the required clinical sample size was N = 30.28 for both groups, following recommendations for ROC-based power analysis [16].

Participants from the clinical group were recruited from the authors’ practices, and references from other mental health specialists in Romania through recruiting announcements made by the authors in the mental health network. For the clinical sample, purposive sampling was chosen to select participants. This non-probability sampling method enabled us to recruit sufficient clinical participants, ensuring adequate statistical power for the primary analyses (criterion validity). All participants from the clinical sample underwent a thorough clinical interview to confirm the ASD diagnosis based on the Diagnostic and Statistical Manual of Mental Disorders, Fifth Edition (DSM-5) diagnostic criteria [7] and their personal and family history. Extensive interviews with the patients’ main caregivers were conducted to confirm the existence of ASD symptoms since childhood. This method was chosen due to the absence of a registry for ASD diagnoses in Romania. All patients included in the clinical sample received their ASD diagnosis at an adult age (18 and over), prior to their inclusion in this study.

The non-clinical sample was selected through social media platforms (WhatsApp: https://www.whatsapp.com, Facebook: https://www.facebook.com, and Instagram: https://www.instagram.com) to allow access to a more diverse population in this study. For the non-clinical sample, a non-probability sampling method was also chosen, namely convenience sampling, mainly due to accessibility reasons.

All study participants were native Romanian speakers. The inclusion criteria for the clinical sample were: individuals aged 18 years or older, a diagnosis of ASD without intellectual and language deficits received at age 18 or later, and confirmation of the diagnosis by the research team. All individuals diagnosed before the age of 18, as well as those younger than 18, were excluded. The inclusion criteria for the general population group consisted of individuals aged 18 and older without a diagnosis of ASD.

Both groups completed a Google Forms questionnaire containing 245 items: informed consent, confidentiality agreement, demographics (age, gender, area, education level, level of contact with mental health specialists, family history of ASD), AQ (50 items), EQ (60 items), and PDSQ (125 items), with a 20 to 25-minute completion time. Data was collected during December 2024–February 2025. The anonymized data set can be found at the following link: https://osf.io/qxrmj/?view_only=5a3c3ccfef2c48c19d54bb7aa796c514.

### 2.2 Methods

The AQ scale is concordant with the Diagnostic and Statistical Manual of Mental Disorders, Fourth Edition, Text Revision (DSM-IV-TR) criteria [17], recording the autism spectrum elements in a 50 item self-report questionnaire analyzing 5 functional characteristics of ASD throughout 10 items each: social abilities impairment; attention disturbance (flexibility, mobility, focus); attention to details; communication impairment; and poor imagination [18]. Scoring is made on a 1–4 Likert scale (1—total agreement, 4—total disagreement), the subject receiving one point on each item if an abnormal score is recorded. To avoid bias, about half of the items were negatively formulated, the other half being positively formulated, corresponding to typical responses for people with a diagnosis of ASD without cognitive and language delay. The AQ score varies between 0 and 50 [18, 19].

The EQ scale consists of 60 items, 40 analyzing empathy and 20 fillers with a score varying between 0 and 80 scored on the same Likert scale as the AQ, with 1 point for average empathy and 2 points for high empathy level [20]. Adults diagnosed with ASD without language and cognitive delay score high in AQ and low in EQ, individuals with ASD having a mean AQ of 35.8 compared to the control group (AQ = 16.4) and a mean EQ of 20.4 compared to 42.1 for the control group [20].

AQ and EQ are screening instruments, designed to assess autistic traits and empathic capacity, respectively. The inverse correlation between them suggests that higher levels of autistic traits (as measured by AQ) are typically associated with lower levels of affective and cognitive empathy (as measured by EQ) [21].

PDSQ is a self-report instrument based on Diagnostic and Statistical Manual of Mental Disorders, Fourth Edition (DSM-IV) [17] designed to screen thirteen of the most frequent psychiatric disorders, consisting of 125 items classified in 13 subscales (one for each disorder), and requiring 15 to 20 minutes to complete [14, 22]. The thirteen disorders screened in PDSQ were classified in 6 categories according to the DSM-IV criteria [17]: eating disorders: bulimia/binge-eating disorder; mood disorders: major depressive disorder (MDD), dysthymia; anxiety disorders: panic disorder (PAN), agoraphobia (AGO), post-traumatic stress disorder (PTSD), OCD, generalized anxiety disorder (GAD), and social phobia (SOC); substance use disorders: alcohol and drug; somatoform disorders: somatization disorder and hypochondriasis, and psychosis (PSY) [23]. Given the fact that DSM-5 takes a dimensional approach rather than a categorial one [7], the original six category classification is not necessary anymore, as PTSD and trauma-related disorders received a different chapter, dysthymia became persistent depressive disorder, and somatization disorder and hypochondriasis became illness anxiety disorder [7], but the questions of the PDSQ are still relevant as they give insight on all the pathologies mentioned above, because the diagnosis criteria have not suffered major changes between DSM-IV and DSM-5 [24]. All the questions of PDSQ are dichotomic scored, every “yes” receiving one point if the item is applicable and every “no” receiving 0 if the item is not applicable. Each of the 13 disorders mentioned above receives a score, and the total score functions as a global indicator of psychopathology. Higher subscale scores for PDSQ suggest a higher likelihood of the corresponding psychiatric condition and may indicate the necessity of further clinical evaluation [23].

### 2.3 Analytic Strategy

To explore potential correlates of the AQ and EQ, we relied on the PDSQ subscales and computed Pearson-correlations in the combined sample. The rationale for using both the clinical and the non-clinical participants is that AQ and EQ are considered screening instruments, thus primarily focused on the general population.

We examined the factorial structure of the AQ to evaluate its structural validity in the combined sample. We conducted a confirmatory factor analysis (CFA) using the diagonally weighted least squares (DWLS) estimation method to do this. The DWLS method is particularly suited for ordinal or dichotomous data, especially when the assumption of normality is not met [25]. To assess model fit, we considered several key indices: the comparative fit index (CFI), the root mean square error of approximation (RMSEA), and the standardized root mean squared residual (SRMR). Additionally, we reported the Tucker–Lewis index (TLI). Interpretation of these indices followed the guidelines of Hu and Bentler [26] and Browne and Cudeck [27], which suggest that CFI and TLI values close to 0.95 or higher indicate a good fit, while RMSEA and SRMR values below 0.08 also suggest an acceptable fit, and 0.09 for smaller sample size in SRMR [28].

To estimate reliability, we reported both Cronbach’s alpha and McDonald’s omega. While Cronbach’s alpha is a widely used measure of internal consistency, McDonald’s omega is considered a more robust alternative, as it is less sensitive to violations of the normality assumption [29].

Criterion validity was deduced by conducting two independent *t*-tests between the clinical and the non-clinical groups regarding the AQ and the EQ scores. Finally, sensitivity and specificity analyses were conducted using the ROC and the AUC. The ROC curve is a widely used plot of an instrument’s sensitivity relative to one minus specificity at each cut-off score (multiple confusion matrices). For interpreting the AUC (which can take values between 0.5 for random performance and 1.0 for perfect performance), we utilized the recommendations of Hanley and McNeil [30], who suggest that a fair diagnostic accuracy can be situated between 0.70 and 0.79, a good accuracy between 0.80 and 0.89 and an excellent accuracy between 0.90 and 1.00. Since AQ and EQ were designed to screen for ASD manifestations in the general population, the cut-off score was determined by favoring sensitivity over specificity.

All the statistical analyses were conducted in R, version 4.4.1 (R Foundation for Statistical Computing, Vienna, Austria) [15].

## 3. Results

### 3.1 Correlations With Age and Psychopathology Measures

Regarding the demographic characteristics of our samples, the age for the clinical group was M = 27.96 (SD = 7.39, MIN = 19, MAX = 49), and for the general group, it was M = 38.98 (SD = 11.61, MIN = 20, MAX = 68). Other relevant data are presented in Table 1 (Yates’ continuity correction for chi-square difference test was applied when the expected frequencies were below 5).

**Table 1.** **Demographic characteristics of participants from the clinical and general groups**.

Demographic characteristic		Clinical group, Freq. (%)	General group, Freq. (%)
Gender			
	Female	14 (45.2%)	62 (55.4%)
	Male	13 (41.9%)	50 (44.6%)
	Nonbinary	4 (12.9%)	-
	Fisher’s exact test (two-sided), *p* = 0.003		
Area			
	Rural	3 (9.7%)	8 (7.1%)
	Urban	28 (90.3%)	104 (92.9%)
	χ^2^ (1) = 0.007, *p* = 0.930		
Education			
	Middle school	-	1 (0.9%)
	High school	12 (38.7%)	14 (12.5%)
	Undergraduate studies	10 (32.3%)	33 (29.5%)
	Graduate studies	8 (25.8%)	52 (46.4 %)
	Doctoral studies	1 (3.2%)	6 (5.4%)
	Post-doctoral studies	-	6 (5.4%)
	Fisher’s exact test (two-sided), *p* = 0.019		
Psychologist referral			
	Yes	30 (96.8%)	63 (56.3%)
	No	1 (3.2%)	49 (43.8%)
	χ^2^ (1) = 17.53, *p* = 0.001		
Psychiatrist referral			
	Yes	29 (93.5%)	28 (25%)
	No	2 (6.5%)	84 (75%)
	χ^2^ (1) = 47.59, *p* < 0.001		
Speech therapist referral			
	Yes	3 (9.7%)	7 (6.3%)
	No	28 (90.3%)	105 (93.8%)
	χ^2^ (1) = 0.069, *p* = 0.791		
Psychiatry inpatient			
	Yes	9 (29%)	4 (3.6%)
	No	22 (71%)	108 (96.4%)
	χ^2^ (1) = 16.08, *p* < 0.001		
Family ASD diagnosis			
	Yes	6 (19.4%)	3 (2.7%)
	No	25 (80.6%)	109 (97.3%)
	χ^2^ (1) = 8.79, *p* = 0.003		
PDSQ scores			
	MDD	Median = 8 (IQR = 4)	Median = 2 (IQR = 8)
	W = 670.5, *p* < 0.001		
	PTSD	Median = 1 (IQR = 2)	Median = 1 (IQR = 8.5)
	W = 1327.5, *p* = 0.036		
	Bulimia	Median = 1 (IQR = 1)	Median = 0 (IQR = 1.5)
	W = 1360.5, *p* = 0.029		
	OCD	Median = 1 (IQR = 0)	Median = 0 (IQR = 3)
	W = 653.0, *p* < 0.001		
	Panic	Median = 0 (IQR = 0)	Median = 0 (IQR = 2)
	W = 1254.5, *p* < 0.001		
	Psychosis	Median = 0 (IQR = 0)	Median = 0 (IQR = 0)
	W = 1514.5, *p* = 0.035		
	Agoraphobia	Median = 2 (IQR = 0)	Median = 0 (IQR = 3)
	W = 614.0, *p* < 0.001		
	Social Phobia	Median = 8 (IQR = 2)	Median = 0 (IQR = 7.5)
	W = 567.0, *p* < 0.001		
	Alcohol Abuse	Median = 0 (IQR = 1)	Median = 0 (IQR = 2)
	W = 1480.0, *p* = 0.116		
	Drug Abuse	Median = 0 (IQR = 0)	Median = 0 (IQR = 0)
	W = 1500.5, *p* = 0.020		
	GAD	Median = 6 (IQR = 4)	Median = 1 (IQR = 5)
	W = 711.5, *p* < 0.001		
	Somatization	Median = 2 (IQR = 1)	Median = 0 (IQR = 3)
	W = 1053.5, *p* < 0.001		
	Hypochondriasis	Median = 0 (IQR = 0)	Median = 0 (IQR = 1)
	W = 1463.5, *p* = 0.050		
	Total	M = 37.77 (SD = 24.10)	M = 12.61 (SD = 12.59)
	t (34.64) = –5.60, *p* < 0.001		
AQ scores		M = 33.61 (SD = 6.14)	M = 16.28 (SD = 6.44)
t (49.83) = –13.76, *p* < 0.001			
EQ scores		M = 26.38 (SD = 10.17)	M = 46.00 (SD = 11.64)
t (53.77) = 9.20, *p* < 0.001			

*Note*. All participants from the clinical group were diagnosed with ASD. ASD, Autism Spectrum Disorder; PDSQ, Psychiatric Diagnosis and Screening Questionnaire; MDD, Major Depressive Disorder; PTSD, Post Traumatic Stress Disorder; OCD, Obsessive-Compulsive Disorder; GAD, Generalized Anxiety Disorder; AQ, Autism Quotient; EQ, Empathy Quotient. The independent samples *t*-test was applied for normally distributed variables across the two groups. The Wilcoxon rank-sum test was applied for highly skewed variables across the two groups.

Regarding the possible associated symptoms of ASD, the strongest correlates of AQ scores in the combined sample (clinical and general) were PTSD, OCD, SOC, and GAD manifestations. In our sample, both clinically diagnosed and non-clinical participants experienced more anxiety-related manifestations as the AQ score increased. Correlation data is presented in Table 2.

**Table 2.** **Correlations between AQ, EQ, and PDSQ in the combined sample**.

	AQ	EQ
MDD	0.476***	–0.266**
PTSD	0.363***	–0.270**
Bulimia	0.212*	–0.073
OCD	0.534***	–0.405***
PAN	0.310***	–0.190*
PSY	0.224**	–0.122
AGO	0.487***	–0.339***
SOC	0.520***	–0.367***
Alcohol	0.153	–0.186*
Drug	0.243**	–0.069
GAD	0.454***	–0.238**
Somatization	0.373***	–0.271**
Hypochondria	0.224**	–0.225**
Total	0.566***	–0.364***

*Note.* Computed correlation used Pearson-method with listwise-deletion. ***p < 0.001, **p < 0.01, *p < 0.05. PAN, Panic Disorder; PSY, Psychosis; AGO, Agoraphobia; SOC, Social Phobia.

We also calculated Pearson’s correlations between AQ, EQ, and age in the combined sample. A significant negative correlation was found between AQ and age (r = –0.21, *p* = 0.010), indicating that older participants tended to have lower scores on AQ, and conversely. However, no such relationship was identified between EQ and age.

### 3.2 Reliability and Validity of the AQ and EQ in the Combined Sample

Both AQ and EQ were highly reliable instruments in our sample. Regardless of the selected group (combined, clinical, and general), both instruments delivered satisfactory internal consistency indicators (well above the threshold of 0.70 for Cronbach’s alpha and McDonald’s omega). Data regarding their reliability is presented in Table 3.

**Table 3.** **Internal consistency indicators for AQ and EQ**.

Measure	Combined sample	Clinical sample	General sample
AQ	α = 0.91	α = 0.86	α = 0.80
	ω = 0.92	ω = 0.89	ω = 0.83
EQ	α = 0.92	α = 0.88	α = 0.89
	ω = 0.94	ω = 0.92	ω = 0.91

Regarding the factorial structure of the AQ, we also tested the 5-factor structure in the combined sample that comprised both clinical and non-clinical participants. The CFA on the five-factor model returned adequate fit indices: CFI = 0.933, TLI = 0.930, RMSEA = 0.066, SRMR = 0.151. Although the SRMR exceeds the conventional threshold of 0.08, this is to be expected given the low sample size and the categorical nature of the AQ items (see Dolfi *et al*., 2025 [31] for an extended discussion regarding the AQ factor structure in the Romanian population). This indicates that when clinical participants are present in the sample, AQ discriminates modestly between social skills, attention switching, attention to detail, communication, and imagination deficits as components of ASD.

Regarding the criterion validity, both AQ and EQ returned significant differences between the clinical and the non-clinical group, indicating their capacity to discriminate autism-related manifestations when the clinical diagnosis is established as a comparison criterion. Regarding the AQ score, a t (49.83) = –13.76, *p* < 0.001 was obtained (M_nonclinical_ = 16.28, M_clinical_ = 33.61). Similar results were obtained when EQ was considered: t (53.77) = 9.20, *p* < 0.001 (M_nonclinical_ = 46.00, M_clinical_ = 26.38).

It is also worth mentioning that, regarding the EQ filler items, no difference was recorded between the clinical group and the non-clinical group, t (50.32) = 1.08, *p* = 0.280.

### 3.3 Sensitivity and Specificity

**Fig. 1.**
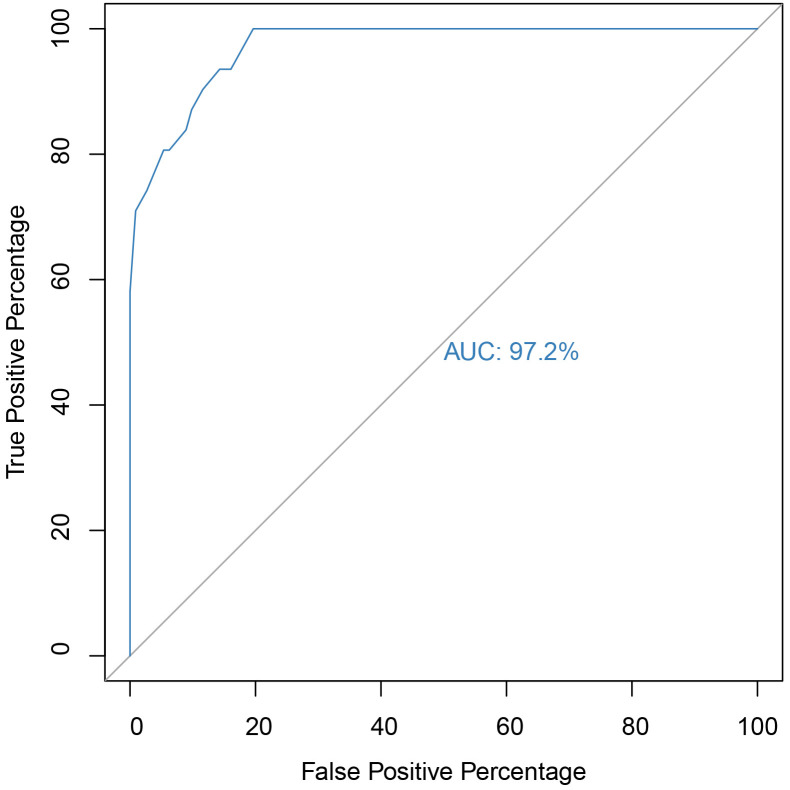
**Receiver operating characteristic (ROC) and area under the curve (AUC) of the AQ scale for identifying ASD cases**. *Note.* 95% CI [0.94, 0.99], *p* < 0.001. CI, confidence interval.

The ROC graph for the AQ scale can be visualized in Fig. 1. The AUC was 97.2%, indicating that, for our sample, AQ had excellent accuracy in detecting ASD cases. Regarding the optimal cut-off score, multiple scores were considered, and subsequent confusion matrices were calculated (see Table 4). The optimal cut-off score that achieved the highest sensitivity and optimal specificity was 21. A cut-off score of 21 for AQ correctly classified 100% of the clinically diagnosed ASD participants as ASD cases (true positives) while correctly identifying 80% of the non-ASD participants as not having ASD (true negatives), with an 84% accuracy, at a 0.06 optimal threshold (Youden’s index = 0.80).

**Table 4.** **Diagnostic statistics for the AQ**.

Cut-off point	Sensitivity	Specificity	Accuracy
21	100%	80%	84%
22	93%	83%	86%
23	93%	85%	87%
24	90%	88%	88%
25	87%	90%	89%
26	83%	91%	89%
27	80%	93%	90%
28	80%	94%	91%
29	74%	97%	92%
30	70%	99%	93%
31	58%	100%	90%

**Fig. 2.**
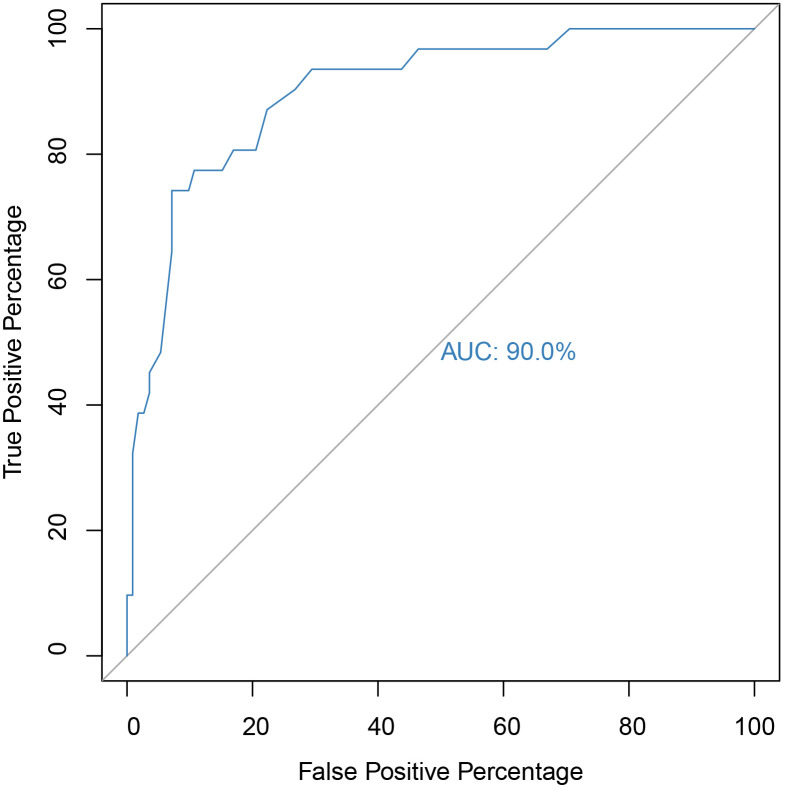
**ROC and AUC of the EQ scale for identifying ASD cases**. *Note.* 95% CI [0.84, 0.96], *p* < 0.001.

The ROC curve for EQ is presented in Fig. 2, with an AUC of 90%, indicating excellent accuracy of AQ in identifying ASD cases within our sample. To determine the optimal cut-off score, various values were evaluated, and confusion matrices were generated (see Table 5). The score of 26 emerged as the most effective, achieving the highest sensitivity while maintaining optimal specificity. At a 0.43 optimal threshold (Youden’s index = 0.67), EQ correctly identified 74% of clinically diagnosed ASD participants as ASD (true positives) and 92% of non-ASD individuals as not having ASD (true negatives), resulting in an overall accuracy of 88%.

**Table 5.** **Diagnostic statistics for the EQ**.

Cut-off point	Sensitivity	Specificity	Accuracy
3	100%	29%	44%
4	96%	33%	46%
5	96%	33%	47%
6	96%	36%	49%
7	96%	37%	50%
8	96%	43%	55%
9	96%	48%	58%
10	96%	53%	62%
11	94%	56%	64%
12	94%	58%	65%
13	94%	59%	67%
14	94%	62%	69%
15	94%	66%	72%
16	94%	70%	75%
17	90%	73%	76%
18	87%	77%	79%
19	83%	78%	79%
20	80%	79%	79%
21	80%	83%	82%
22	77%	84%	83%
23	77%	87%	85%
24	77%	89%	86%
25	74%	90%	86%
26	74%	92%	88%
27	64%	92%	86%
28	48%	94%	84%
29	45%	96%	85%
30	41%	96%	84%
31	38%	97%	84%
33	38%	98%	85%
34	32%	99%	84%
36	25%	99%	83%
37	19%	99%	81%
38	9%	99%	79%
41	9%	100%	80%

## 4. Discussion

This is the first study on the Romanian population which established a cut-off score for the Romanian versions of AQ (50 item version) and EQ (60 items). The cut-off score obtained for AQ for the study sample was 21 and it correctly classified 100% of the clinically diagnosed ASD participants as ASD cases (true positives) while correctly identifying 80% of the non-ASD participants as not having ASD (true negatives), with an 84% accuracy. This is smaller than the original cut-off score of 32 obtained by Baron-Cohen *et al*. [18]; a later study conducted by Austin [32] obtained a cut-off score of 30 on adults. The closest value to our cut-off score of 21 was obtained on the validation of AQ in the French-Canadian population, which had a cut-off score of 22 [33]. The authors of the French–Canadian validation study observed that a lower cut-off score for AQ was best in differentiating individuals with autistic traits from the general population [33], a fact confirmed by our study. The differences in cut-off score among different populations could be due to demographic diversity, the translation process, and cultural factors.

The cut-off score obtained for EQ in the current study is 26, slightly lower compared with the cut-off scores of 30 obtained on the British population [20] and 33 on the French-Canadian population [33]. At the score of 26 it correctly identified 74% of clinically diagnosed ASD participants as ASD (true positives) and 92% of non-ASD individuals as not having ASD (true negatives), resulting in an overall accuracy of 88%.

We also need to discuss that the AQ and EQ questionnaires, based on the DSM-IV diagnostic criteria, were originally introduced in 2001 [18], and have undergone no modifications to date. Since then, the diagnosis criteria have changed, and factors related to culture, population diversity and mobility need to be considered. Also, the public opinion upon neurodivergence has modified in both the population and the scientific community, while AQ and EQ still remain in the same form since their introduction. Despite all this, they remain the most used screening scales for ASD without intellectual and language deficit in adults and are translated in many languages. The studies discussed before, along with our study, confirm the high sensitivity and specificity of the two scales, which proves the fact that these two questionnaires are excellent screening methods for ASD, but taking into consideration the differences in cut-off score obtained on different populations and the demographic diversity, more studies are necessary in order to adapt these two instruments to comply with the modifications underwent both by the diagnostic criteria and by the population in the last decade.

Regarding the potential associated symptoms of ASD, we identified a correlation of the AQ scores in the combined sample (clinical and general) for PTSD, OCD, SOC, and GAD, with both groups scoring higher on manifestations of anxiety as the AQ score increased. Anxiety is a comorbidity frequently associated with ASD [6, 7], but no diagnostic hypothesis can be formulated, as PDSQ is a screening scale. We correlated the AQ score with age and found that it decreases as age increases, a fact supported by the literature [34], but found no correlation between age and EQ.

The main limitation of the present study arises firstly from the fact that the clinical group was very small (32 participants, one being eliminated), which might have impacted the cut-off score results we obtained for AQ and EQ. Also, due to the small group size, gender could not be considered a covariate variable in the main analysis so that no significant statistical analysis could be conducted. For those reasons, the need for future studies with a higher clinical population is in order to better assess cut-off scores for the Romanian population and identify specific cut-off scores per gender. The second limitation is the fact that the PDSQ scale has many clinical subscales (13, one for each disorder screened) and is dichotomic and self-administered, with “yes”/“no” answers, catching either fully developed clinical symptomatology, or none. This could lead to missing an entire range of subclinical and low to moderate intensity symptomatology, hence the weak correlations we obtained between ASD and other associated symptoms in this study. Finally, another limitation of the present study is that only screening scales were used, hence no diagnostic hypotheses could be formulated. Future studies are necessary to prove stronger correlations between ASD and other pathologies in Romania, especially in correlation with a specific pathology and symptom severity.

## 5. Conclusion

Symptoms of PTSD, OCD, SOC, and GAD are correlated with a higher AQ score, hence more studies are needed to assess the associated symptoms and comorbidities of ASD for the Romanian population.

AQ and EQ have proven to be excellent screening instruments in the Romanian sample, with excellent sensitivity and specificity in identifying individuals with autistic traits. However, to obtain a diagnosis of ASD, a thorough evaluation, which includes a clinical interview, personal and family history, and, if possible, an interview with the primary caregiver, is necessary.

## Availability of Data and Materials

The anonymized data set can be found in an online data repository at the following link: https://osf.io/qxrmj/?view_only=5a3c3ccfef2c48c19d54bb7aa796c514.
